# Effectiveness and Safety of Polatuzumab Vedotin Plus an Anti‐CD20 Monoclonal Antibody (Rituximab or Obinutuzumab) and Zanubrutinib in Relapsed/Refractory Diffuse Large B‐Cell Lymphoma

**DOI:** 10.1002/cam4.71162

**Published:** 2025-08-19

**Authors:** Yian Zhang, Yuhong Ren, Jingli Zhuang, Ling Yuan, Zhimei Wang, Xuejiao Zhang, Weiguang Wang, Zhixiang Cheng, Luya Cheng, Jing Li, Peng Liu

**Affiliations:** ^1^ Department of Hematology, Zhongshan Hospital Fudan University Shanghai China; ^2^ Cancer Center, Zhongshan Hospital Fudan University Shanghai China

**Keywords:** diffuse large B‐cell lymphoma, effectiveness, Polatuzumab vedotin, relapsed/refractory, safety

## Abstract

**Background:**

Approximately 30% of patients with diffuse large B‐cell lymphoma (DLBCL) relapse or are refractory to first‐line treatment. This study aimed to determine the effectiveness and tolerability of the combination of Polatuzumab vedotin and Zanubrutinib plus Rituximab (Pola‐ZR) or Obinutuzumab (Pola‐ZG) in patients with relapsed/refractory (R/R) DLBCL.

**Methods:**

We conducted a prospective observational study as part of our registered cohort study (NCT06203652). Patients were planned to receive six 21‐day cycles of Pola‐ZR/G, followed by Zanubrutinib monotherapy. The primary endpoint was to evaluate the best overall response rate (BOR), while secondary endpoints included the median progression‐free survival (mPFS), safety, and complete response rate (CRR). We collected data from 73 R/R DLBCL patients who received traditional salvage therapies (TST). After propensity score matching, they were paired 1:1 with the Pola‐ZR/G arm to compare the effectiveness and survival outcomes.

**Results:**

Twenty‐two patients were enrolled (median age 68 years) in the Pola‐ZR/G group. After a median follow‐up of 16.1 months, the investigators assessed BOR; it was 70% (77.77% for Pola‐ZR and 63.6% for Pola‐ZG cohort), and CRR was 45% in 20 evaluable R/R patients. The mPFS was 8.3 months and was higher compared to the TST cohort. The median overall survival (OS) of the Pola‐ZR/G cohort had not been reached at the time of analysis. The most common grade 3 to 4 adverse events were infections of all types and hematological toxicity.

**Conclusion:**

In our study, patients derived clinical benefit after receiving Pola‐ZR/G compared to TST; the regimens had a tolerable safety profile in patients with R/R DLBCL.

## Graphical abstract

Patients underwent a mid‐term evaluation after completing 3 cycles of Pola‐ZR/G, followed by an evaluation at the end of 6 cycles. Additionally, we collected data from 73 R/R DLBCL patients who received traditional salvage therapies (TST). Following propensity score matching, patients receiving Pola‐ZR/G or TST were paired 1:1, with 20 patients selected from each treatment arm for effectiveness comparison.

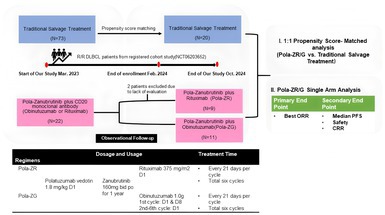

**Abbreviations**
ASCT: autologous stem cell transplantation
BOR: best overall response rate
BTKi: Bruton's tyrosine kinase inhibitor
CIs: confidence intervals
COO: cell of origin
CRR: complete response rate
CTCAE: Common Terminology Criteria for Adverse Events
DLBCL: diffuse large B‐cell lymphoma
DOR: duration of overall response
GCB: germinal center B‐cell like
IPI: international prognostic index
mPFS: median progression‐free survival
Pola: Polatuzumab vedotin
Pola‐ZG: Polatuzumab vedotin and Zanubrutinib plus Obinutuzumab
Pola‐ZR: Polatuzumab vedotin and Zanubrutinib plus Rituximab
PS: performance status
PSM: propensity score matching
R/R: refractory/relapsed
TST: traditional salvage therapies

## 1 Introduction

Diffuse large B‐cell lymphoma (DLBCL) is a heterogeneous and aggressive lymphoma. In clinical practice, appropriate treatment regimens are generally selected based on the immune and molecular phenotypic characteristics [1]. Unfortunately, about 30% of patients are difficult to cure or relapse after R‐CHOP treatment, known as relapsed/refractory (R/R) DLBCL. High‐dose chemotherapy followed by autologous stem cell rescue (HDT‐ASCR) is the primary treatment option for chemotherapy‐sensitive and transplant‐eligible patients [2, 3]. However, patients who are not suitable or have failed HDT‐ASCR rely more on new drugs and innovative treatment plans.

In recent years, the emergence of novel monoclonal antibodies [4, 5, 6, 7], immune modulators [8], small molecular inhibitors [9] and the chimeric antigen receptor (CAR) T‐cell therapy [10] has made it possible to treat B‐cell lymphoma without chemotherapy. Polatuzumab vedotin (Pola), a CD79b antibody‐drug conjugate, has demonstrated significant clinical efficacy in R/R DLBCL patients. With the novel triplet combination of Pola, rituximab, and lenalidomide, it achieved an end‐of‐induction objective response rate (ORR) of 39% (90% CI, 27–52) and complete response rate (CRR) of 31% (90% CI, 20–43) [11]. The combination of Pola, Bendamustine, and rituximab (Pola‐BR) has been shown to significantly prolong the survival of R/R DLBCL patients, with a median PFS (mPFS) of 9.5 (6.2–13.9) months compared to 3.7 (2.1–4.5) months for BR [12].

In our study, we conducted a real‐world prospective observational study of R/R DLBCL patients as part of our registered cohort study (The Pathogenesis and Prognostic Factors of Lymphoma, NCT06203652). Our objective was to evaluate the effectiveness and safety of Pola‐ZR (Polatuzumab vedotin, Zanubrutinib and Rituximab) or Pola‐ZG (Polatuzumab vedotin, Zanubrutinib and Obinutuzumab) as potential new treatment options for R/R DLBCL patients.

## 2 Methods

### 2.1 Patients and Study Design

**FIGURE 1:**
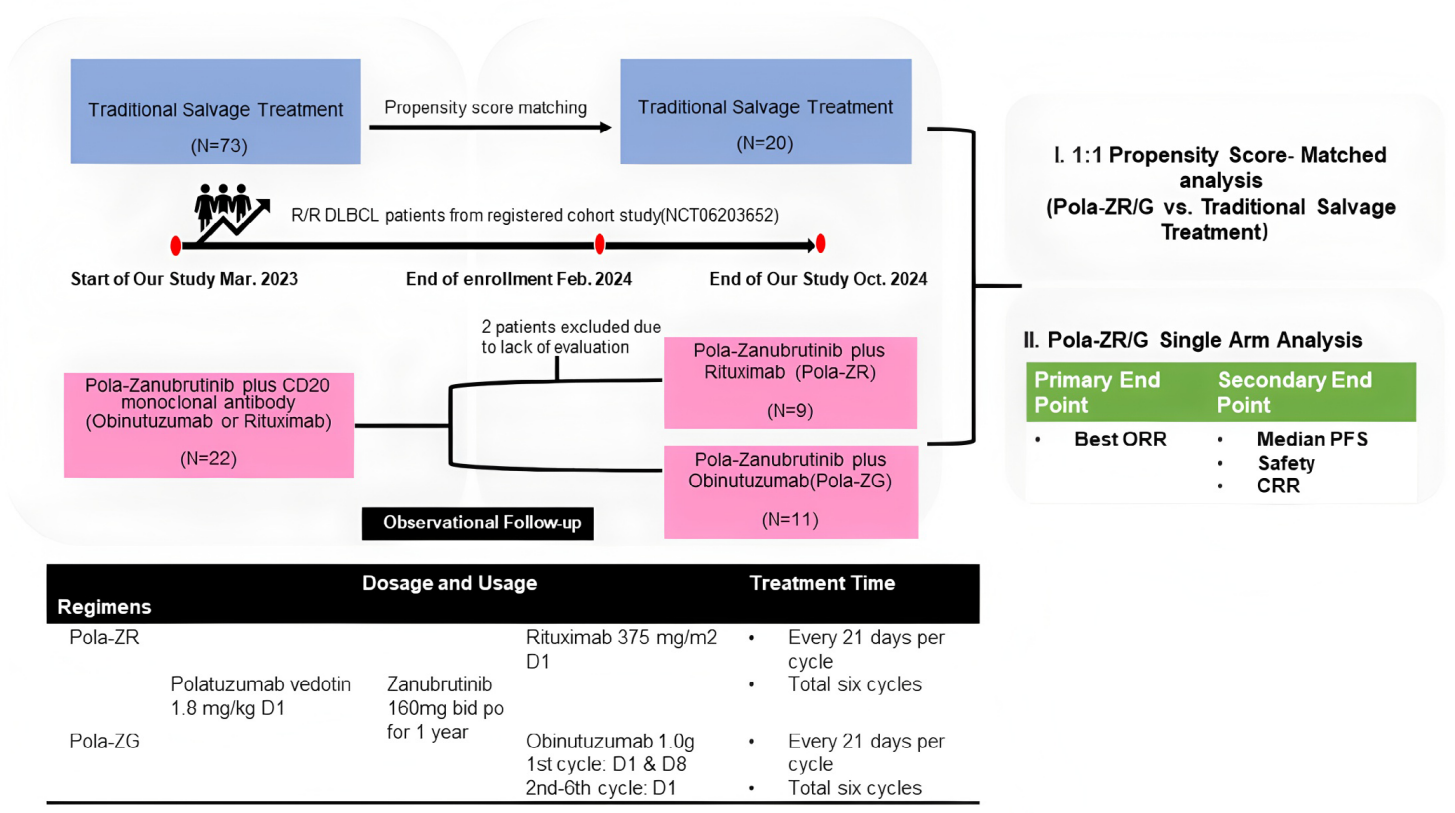
Study schema. BOR, best overall response rate; CRR, complete response rate; ORR, overall response rate; PFS, progression‐free survival.

We enrolled 22 R/R DLBCL patients at our center between April 1, 2023, and January 31, 2024. All the patients confirmed a histopathological diagnosis of DLBCL and had received at least one previous line of treatment. Eleven patients were assigned to each of the two regimens (Pola‐ZR and Pola‐ZG). Informed consent for participation and biological sample donation was obtained from each patient. This study was conducted in accordance with the ethical principles of the Declaration of Helsinki. Patients underwent a mid‐term evaluation after completing 3 cycles of Pola‐ZR/G, followed by an evaluation at the end of 6 cycles and the initiation of Zanubrutinib 160 mg bid monotherapy for 6 months. The overall duration for Zanubrutinib oral medication is about 10 months. Seven patients received consolidation with CAR‐T cell therapy (3 patients) or autologous transplant (4 patients). Additionally, we collected data from 73 R/R DLBCL patients who received traditional salvage therapies (TST) between the year 2022 and 2023. Following propensity score matching, patients receiving Pola‐ZR/G or TST were paired 1:1 to adjust for clinical characteristics, with 20 patients selected from each treatment arm for effectiveness comparison (Figure 1).

### 2.2 Clinical Assessments

Laboratory assessments and adverse events (AEs) were monitored throughout the study. AEs were reported according to the Common Terminology Criteria for Adverse Events (CTCAE) v4.024. Response assessments were conducted by investigators based on PET‐CT or enhanced CT scans, utilizing the modified 2014 Lugano criteria. Doctors determine which modality (PET‐CT or enhanced CT) to utilize based on the diagnostic tests selected. Refractory status was defined by stable or progressive disease after the last regimen. Response assessments were required before cycle 4 (interim evaluation), at the end of 6 cycles, and then every 3 months until disease progression.

The primary endpoint was the evaluation of the best ORR (BOR), defined as the best response of complete response or partial response at any time during the study. Secondary endpoints included mPFS, safety, and CRR at the end of Pola‐ZR/G treatment. PFS was defined as the time from the initiation of the treatment to the earliest occurrence of disease progression, relapse, or death as a result of any cause in patients with DLBCL. Patients without disease progression, relapse, or death were censored at the time of the last assessment.

### 2.3 Statistical Analyses

Categorical variables related to clinical characteristics were reported as counts and percentages, while continuous variables were expressed as median (range). Differences between groups were assessed using the *χ* ^2^ test or Fisher's exact test as appropriate. A *p* value of < 0.05 indicated statistically significant, and all reported *p* values are 2 sided. Safety analyses included all patients receiving ≥ 1 dose of Pola‐Z/G, while effectiveness analyses included all patients receiving ≥ 1 dose of Pola‐Z/G also with imaging evaluation results. Survival was estimated using the Kaplan–Meier method with 95% confidence intervals (CIs). To compare the efficiency and safety between Pola‐ZR/G and TST regimens while minimizing potential confounders and selection bias, we employed 1:1 propensity score matching (PSM) using the nearest‐neighbor method with a caliper size of 0.05 based on logistic regression analysis. The Cox proportional hazards model was utilized for multivariate regression analysis. All analyses were performed with data obtained until February 2024, using SPSS software (version 22.00).

## 3 Results

### 3.1 Patients and Treatment Exposure

Data collection concluded on February 1, 2024, with further follow‐up continuing until October 31, 2024. The median age of the 22 patients enrolled was 68 years, with 68% having a Performance Score (PS) ≥ 2 points. The majority of patients were classified as Ann Arbor stage III‐IV, with an IPI score of 4–5 points. More than one‐third of patients presented with bulky disease prior to treatment. All the patients underwent fluorescence in situ hybridization (FISH) testing; only two patients were diagnosed with double‐hit lymphoma. Immunohistochemical COO typing showed that there were 9 patients with GCB subtype and 13 patients with non‐GCB subtype. Due to a higher number of patients with the cell of origin (COO) subtype of non‐GCB (13/22), the proportion of patients exhibiting double expression in the study exceeded 30% (7/22). In Pola‐ZR and Pola‐ZG subgroups, the median number of prior lines of treatment was one (range, 1–5), with more than half of the patients receiving Pola‐ZR/G as a second‐line salvage treatment option. 18.18% of patients had previously received Bendamustine‐based combinations, while 40.91% had been treated with lenalidomide (Table 1).

**TABLE 1:** Baseline characteristics of Pola‐ZR/G patients.

	Total (*n* = 22)	Pola‐ZR (*n* = 11)	Pola‐ZG (*n* = 11)
Median age, years	68	67	76
Age ≥ 70, *n* (%)	10 (45.45)	4 (36.36)	6 (54.54)
Male, *n* (%)	13 (59.09)	6 (54.54)	7 (63.63)
PS ≥ 2, *n* (%)	15 (68.18)	9 (81.81)	6 (54.54)
Ann Arbor stage, *n* (%)			
I–II	3 (13.64)	1 (9.09)	2 (18.18)
≥ III	19 (86.36)	10 (91.67)	9 (81.81)
IPI score, *n* (%)			
0–1	2 (9.09)	0 (0)	2 (18.18)
2–3	8 (36.36)	5 (45.45)	3 (27.27)
4–5	12 (54.55)	6 (54.55)	6 (54.55)
COO, *n* (%)			
GCB	9 (40.91)	3 (27.27)	6 (54.55)
nonGCB	13 (59.09)	8 (72.73)	5 (45.45)
Bulky disease (≥ 7 cm), *n* (%)	9 (40.91)	3 (27.27)	6 (54.54)
Cytogenetics, *n* (%)			
Double hit lymphoma	2 (9.09)	0 (0)	2 (18.18)
Double expression lymphoma	7 (31.82)	5 (45.45)	2 (18.18)
Bone marrow involvement, *n* (%)	3 (13.64)	1 (9.09)	2 (18.18)
Prior lines received, *n* (%)			
I‐II	12 (54.55)	6 (54.55)	6 (54.55)
≥ III	10 (45.45)	5 (45.45)	5 (45.45)
Prior regimens received, *n* (%)			
R‐CHOP or R‐miniCHOP	18 (81.82)	8 (72.73)	10 (90.91)
R‐DA‐EPOCH	3 (13.64)	1 (9.09)	2 (18.18)
Gemox	7 (31.82)	5 (45.45)	2 (18.18)
Bendamustine	4 (18.18)	1 (9.09)	3 (27.27)
Lenalidomide	9 (40.91)	7 (63.64)	2 (18.18)
Zanubrutinib	1 (4.55)	1 (9.09)	0 (0)
Clinical trial	2 (9.09)	1 (9.09)	1 (9.09)

Abbreviations: COO, cell of origin; GCB, germinal center B‐cell like; IPI, international prognostic index; Pola‐ZG, Polatuzumab vedotin, Zanubrutinib and Obinutuzumab; Pola‐ZR, Polatuzumab vedotin, Zanubrutinib and Rituximab; PS, performance score.

**FIGURE 2:**
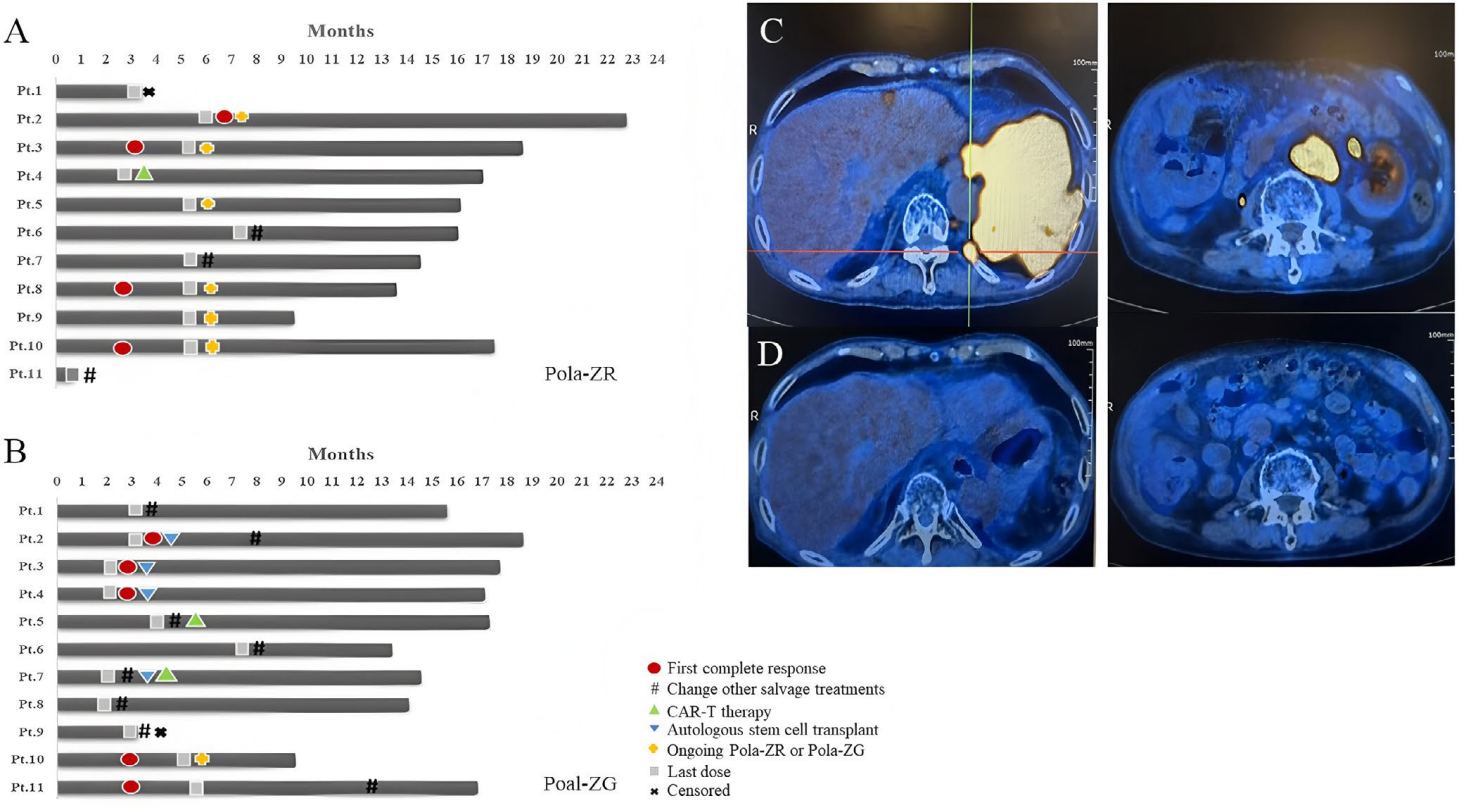
Timelines of 22 patients' clinical courses of treatment and evaluation. Swimmer plot of the clinical course of 22 patients who received Pola‐ZR (A) or Pola‐ZG (B). Images of the PET‐CT scans of a 78‐year‐old male R/R DLBCL patient (Ann Arbor stage IV, IPI score 5 points, nonGCB type) at baseline (C) and after three cycles of Pola‐ZG (D). He received R‐miniCHOP as a first‐line therapy and R2 treatment after first‐line treatment failure. He received the Pola‐ZG regimen on January 19, 2024. The patient achieved complete metabolic remission after three cycles of Pola‐ZG.

Overall, the median number of treatment cycles was 5 for all the 22 patients. There are 64% (7 of 11) of patients in the Pola‐ZR subgroup and 36% (4 of 11) in the Pola‐ZG subgroup who completed 6 cycles of the planned regimens. Patients in the Pola‐ZR subgroup received a median of 6 cycles (range, 1–6), while Pola‐ZG patients received a median of 3 cycles (range, 1–6). All patients received 100% dose intensity of Polatuzumab (1.8 mg/kg), Zanubrutinib (160 mg bid), and the standard dose of CD20 monoclonal antibody (Rituximab 375 mg/m^2^ or Obinutuzumab 1.0 g). The median treatment interval for these patients was 21.2 days, with an average of 22.7 days, indicating good tolerance to both treatment regimens (Figure 2). Two patients were excluded from the efficacy evaluation due to the lack of efficacy assessment results (refuse to return to the hospital for mid‐term evaluation). All patients had undergone safety evaluation.

### 3.2 Safety of Pola‐ZR/G

The main drug‐related AEs associated with the Pola‐ZR/G combinations included infections (40.91%), with a higher incidence in patients receiving Pola‐ZG compared to Pola‐ZR (45.45% vs. 36.36%). The predominant type of infection was lung infection, occurring in 6 of the 9 patients with any infection. Half of the patients with lung infection experienced Grade 3 AEs, requiring intravenous antibiotics. Infections may arise during any treatment cycle or following completion of 6 cycles, including bacterial pathogens such as *Streptococcus pneumoniae* and *Haemophilus influenzae* ; viral agents like cytomegalovirus, herpesviruses, and reactivated hepatitis viruses; along with fungal infections such as pulmonary aspergillosis and Pneumocystis jirovecii pneumonia (PJP). Prophylactic strategies include levofloxacin for bacterial prevention during treatment‐induced agranulocytosis and acyclovir for herpesvirus reactivation prophylaxis. Trimethoprim‐sulfamethoxazole (TMP‐SMX) serves as first‐line prevention for PJP. Regarding hematological toxicity, leukopenia and neutropenia each occur at 31.82% incidence, with thrombocytopenia less frequent (18.18%). Granulocyte colony‐stimulating factor (G‐CSF) is administered when neutrophils fall below 1.0 × 10^9^/L. Zanubrutinib requires suspension for either Grade ≥ 3 neutropenia concurrent with infection/fever, or Grade 4 hematological toxicity. Infusion reactions occurred in 13.64% of patients, mainly manifested as fever (9.09%) and rash (4.55%), with no reported headaches. No serious grade 3–4 infusion reactions were recorded during our study. Three patients experienced liver dysfunction, all of whom received Pola‐ZR. Three patients experienced grade 1–2 hyperuricemia during treatment, potentially related to the oncolytic response caused by high tumor burden. These cases were managed effectively with hydration, alkalization, and diuresis. Gastrointestinal AEs include diarrhea (9.09%) and nausea (13.64%), with no patients reporting constipation. Two patients receiving Pola‐ZG reported joint and bone pain, both of which were grade 1–2 and relieved after the first cycle. A total of three patients reported drug‐related peripheral neuropathy, including burning, numbness, tingling, or pain in the arms, fingers, legs, or feet. All the patients with peripheral neuropathy were Grade 1‐2, not interfering with function or activities of daily living. Overall, after reviewing safety data, no patient was permanently discontinued due to AEs (Table 2).

**TABLE 2:** Summary of adverse events.

*N* (%)	Total (*n* = 22)		Pola‐ZR (*n* = 11)		Pola‐ZG (*n* = 11)	
	All AE	Grade 3–4	All AE	Grade 3–4	All AE	Grade 3–4
Infections of all kind	9 (40.91)	3 (13.64)	4 (36.36)	2 (18.18)	5 (45.45)	1 (9.09)
Neutropenia	7 (31.82)	3 (13.64)	4 (36.36)	2 (18.18)	3 (27.27)	1 (9.09)
Thrombocytopenia	4 (18.18)	0 (0)	0 (0)	0 (0)	4 (36.36)	0 (0)
Anemia	13 (59.09)	1 (4.55)	5 (45.45)	0 (0)	8 (72.73)	1 (9.09)
Leukopenia	7 (31.82)	2 (9.09)	4 (36.36)	2 (18.18)	3 (27.27)	0 (0)
Infusion‐related reaction	3 (13.64)	0 (0)	2 (18.18)	0 (0)	1 (9.09)	0 (0)
Pyrexia	2 (9.09)	0 (0)	2 (18.18)	0 (0)	0 (0)	0 (0)
Rash	1 (4.55)	0 (0)	0 (0)	0 (0)	1 (9.09)	0 (0)
Hypotension	0 (0)	0 (0)	0 (0)	0 (0)	0 (0)	0 (0)
Hyperuricemia	3 (13.64)	0 (0)	3 (27.27)	0 (0)	0 (0)	0 (0)
Liver disfunction	3 (13.64)	2 (9.09)	3 (27.27)	2 (18.18)	0 (0)	0 (0)
Diarrhea	2 (9.09)	0 (0)	2 (18.18)	0 (0)	0 (0)	0 (0)
Nausea	3 (13.64)	0 (0)	2 (18.18)	0 (0)	1 (9.09)	0 (0)
Asthenia	4 (18.18)	0 (0)	2 (18.18)	0 (0)	2 (18.18)	0 (0)
Headache	0 (0)	0 (0)	0 (0)	0 (0)	0 (0)	0 (0)
Constipation	0 (0)	0 (0)	0 (0)	0 (0)	0 (0)	0 (0)
Arthralgia	2 (9.09)	0 (0)	0 (0)	0 (0)	2 (18.18)	0 (0)
Peripheral neuropathy	3 (13.64)	0 (0)	2 (18.18)	0 (0)	1 (9.09)	0 (0)

Abbreviations: Pola‐ZG, Polatuzumab vedotin, Zanubrutinib and Obinutuzumab; Pola‐ZR, Polatuzumab vedotin, Zanubrutinib and Rituximab.

### 3.3 Effectiveness of Pola‐ZR/G

The median prospective follow‐up time was 16.1 months. The BOR as assessed by investigators was 70% (14/20), with 77.77% for Pola‐ZR and 63.6% for Pola‐ZG. CRR was 45% (9 of 20) across all patients, including 44.44% for Pola‐ZR and 45.45% for Pola‐ZG (Figure S1–S4). For patients who experienced first‐line progression and received Pola‐ZR or Pola‐ZG as a second‐line salvage treatment, the overall BOR was 60%, with a CRR of 41.67%. In cases treated as ≥ 3rd line, BOR was 75%, and the CRR reached 50% (Table S1–S4). Responses were consistent regardless of cytogenetic status (double expression or double hit lymphoma), COO subtype (GCB or nonGCB), tumor burden, and previous treatment lines. Among the 14 responsive patients, the median response time was 64 days, and the median duration of overall response (DOR) was 10.35 months. The median time to achieve CR was 64.5 days (about 3 cycles).

### 3.4 Characteristics, Response and Survival After PSM

After PSM (1:1) to adjust for clinical characteristics, 1. There were no statistical differences in age ≥ 70, gender, performance status (PS) score, Ann Arbor stage, international prognostic index (IPI) score, COO subtypes, bulky diseases, and cytogenetics (Table S2). In the TST arm, 42.47% (31/73) patients received Gemox‐based regimens, 28.77% (21/73) received ICE‐based regimens, and 16% (5/73) received GDP‐based regimens. About 16% (5/73) patients chose chemo‐free regimens, while a small number of patients (2.7%, 2/73) received high‐intensity regimens such as DA‐EPOCH. After PSM, among the TST arm, 45% (9/20) patients received Gemox‐based regimen, 20% (4/20) received ICE‐based regimen, and about 10% (2/20) chose chemo‐free regimens, while 5% opted for high‐intensity regimens.

**FIGURE 3:**
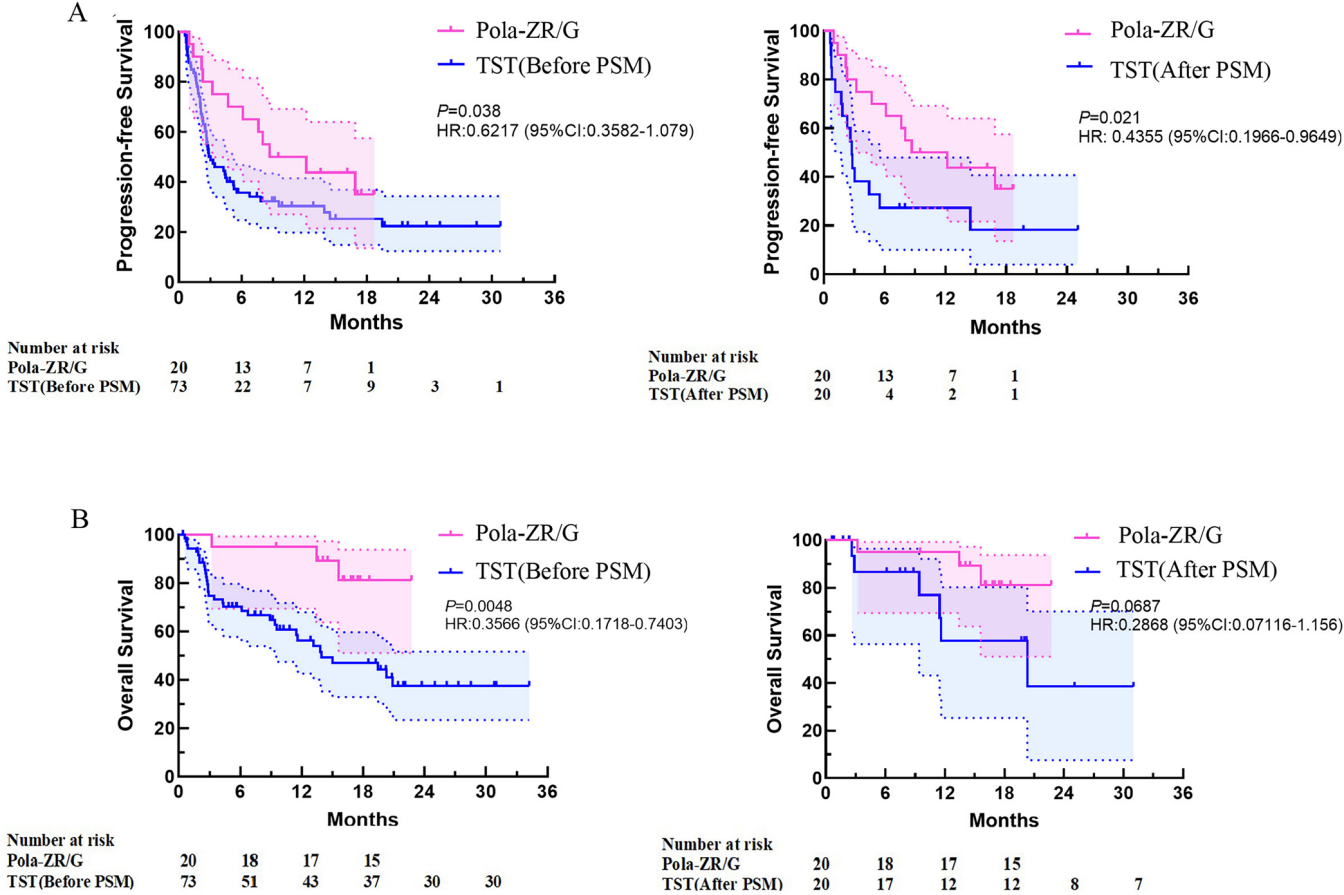
Kaplan–Meier analysis of progression‐free survival (A) and overall survival (B) in patients before (left) and after (right) 1:1 propensity score matching. 95% CI, 95% confidential interval; HR, hazard ratio; Pola‐ZG, Polatuzumab vedotin, Zanubrutinib, and Obinutuzumab; Pola‐ZR, Polatuzumab vedotin, Zanubrutinib, and Rituximab; PSM, propensity score matching; TST, traditional salvage therapies.

Overall, BOR and CRR were higher for patients from the Pola‐ZR/G arm compared to those in the TST arm (both before and after PSM) (Table S3). After a median follow‐up of 16.1 (3.2–22.7) months, PFS and OS were significantly improved with Pola‐ZR/G compared to TST. At study completion, the mPFS was 8.7 months (95% CI 0.577–16.823). Consistent benefits in risk reduction were observed for PFS before (HR, 0.6217; 95% CI, 0.3582–1.079; *p* = 0.038) and after (HR, 0.4355; 95% CI, 0.1966–0.9649; *p* = 0.021) PSM (Figure 3). The median OS was not reached for the Pola‐ZR/G arm. OS was significantly improved in the Pola‐ZR/G arm before PSM, with the risk of death reduced by 64% (HR, 0.3565; 95% CI, 0.1718–0.7403; *p* < 0.01) compared to TST (median OS, 13.9 months; 95% CI, 5.24–22.92 months). The difference in OS after PSM did not reach statistical significance between the two groups.

**FIGURE 4:**
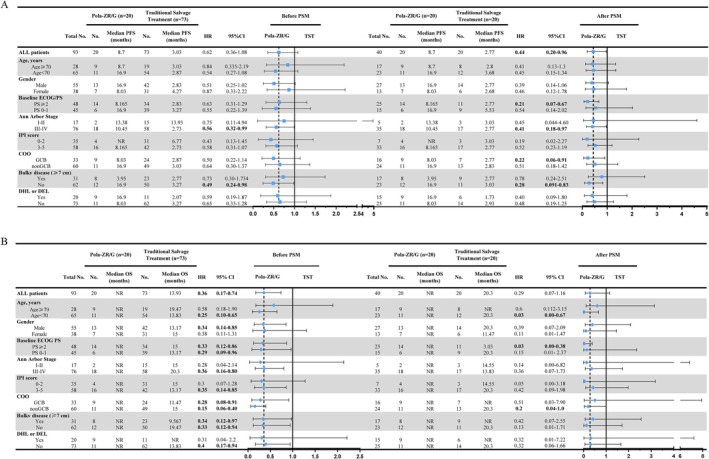
Forest plots of risk factors for survival before and after PSM using multivariable logistic regression analysis. Forest plot of PFS (A) and OS (B) according to clinical and biologic characteristics. COO, cell of origin; DEL, double expression lymphoma; DHL, double hit lymphoma; NR, not reached; Pola‐ZG, Polatuzumab vedotin, Zanubrutinib, and Obinutuzumab; Pola‐ZR, Polatuzumab vedotin, Zanubrutinib, and Rituximab; TST, traditional salvage therapies.

Regarding the forest plots of PFS according to clinical and biologic characteristics, patients benefited from the Pola‐ZR/G regimen, especially for those with Eastern Cooperative Oncology Group (ECOG) ≥ 2, Ann Arbor III‐IV, GCB subtype, and patients without bulky disease. For the forest plots of OS, patients benefiting from Pola‐ZR/G included those age < 70, ECOG ≥ 2, nonGCB subtype (Figure 4). The difference between OS and PFS in subgroup forest plot analysis of risk factors may be related to the small sample size after PSM, which also prevents the establishment of statistical significance for some characteristics.

## 4 Discussion

In our 1:1 PSM comparison study, treatment with Pola‐ZR/G resulted in a significantly improved BOR, CRR, PFS, and OS compared to TST. To our knowledge, this is the first study on R/R DLBCL treated with the novel triplet combination of Pola, Rituximab/Obinutuzumab, and Zanubrutinib. Since this is an observational study, there were no restrictions on which patients receive the Pola‐ZR or Pola‐ZG regimen. Doctors typically discuss therapy alternatives with their patients before making a decision. Patients' choices, on the other hand, may be driven more by financial concerns, such as insurance coverage.

Pola received accelerated approval from the FDA on June 10, 2019, for use in combination with Bendamustine and a Rituximab product for adult patients with R/R DLBCL after at least two prior therapies. Studies, such as GO29365 (NCT02257567) [12, 13], highlight the potential of Pola‐based regimens in improving outcomes for patients with R/R DLBCL Natsumi Kawasaki et al. discovered that Pola increased the expression of CD20 and sensitized Pola‐refractory cells to Rituximab‐induced complement‐dependent cytotoxicity (CDC) [14]. Meanwhile, the combination of Bruton's tyrosine kinase inhibitor (BTKi) with anti‐CD20 therapy has been utilized in many B‐cell lymphomas [15, 16]. It has been proposed that combining these two drugs will target and eliminate blood lymphocytes, thereby shortening the time to response in B‐cell lymphoma patients [17]. Zanubrutinib has stronger selectivity and fewer off‐target effects on BTK than first‐generation BTK inhibitors [18, 19, 20]. Obinutuzumab is a glycoengineered humanized type II anti‐CD20 monoclonal antibody, exerting its anti‐tumor activity through direct induction of cell death and ADCC [21]. Low expression or loss of CD20, which causes resistance to rituximab, does not significantly correlate with the killing effect mediated by ADCC. This difference enables Obinutuzumab to overcome the resistance mechanism or insufficient response associated with Rituximab. Nonetheless, Pola‐ZR/G still provides a trusted result on survival improvement for Pola‐Rituximab/Obinutuzumab‐Zanubrutinib combination therapy in treating R/R DLBCL patients.

Table S4 summarizes recent studies on R/R DLBCL treatment in several countries, including Pola‐based regimens (Pola‐R2 [11] and Pola‐BR [13]), CAR‐T cell therapy [22] (Zuma‐7 study), and Pola combined with traditional chemotherapies [23]. Although cross‐trial comparisons are challenging due to inconsistent response criteria and variations in patients' baseline characteristics, we found that the combination of Pola‐ZR/G showed good effects and tolerability in a high‐risk population (median age close to 70 years; 68% of the patients had a PS score ≥ 2; the majority of patients were Ann Arbor stage III‐IV with an IPI score ≥ 4; more than one‐third of patients have bulky disease before treatment). Before PSM, the median age, proportion of patients with a high‐risk genetic background, and number of previous treatment lines in the Pola‐ZR/G group were higher than in the TST group. It appears that Pola‐ZR/G could potentially demonstrate some survival advantage before PSM; however, further investigation would be needed to confirm this observation. Our study found that Pola‐ZR/G may be an effective treatment option for R/R DLBCL patients who have failed standard first‐line treatment, particularly those with Ann Arbor III‐IV, ECOG ≥ 2 or frail patients who cannot tolerate standard‐dose chemotherapy due to hematological toxicity (e.g., need blood transfusion, treatment delay, or dosage reduction). Recently, CD20xCD3 bispecific antibodies demonstrate significant efficacy in R/R DLBCL. As fixed‐cycle monotherapy, they induce durable remissions in patients with ≥ 2 prior lines of therapy [7]. The STARGLO study revealed that combining one such antibody with gemcitabine and oxaliplatin (GEMOX) improved OS and PFS in transplant‐ineligible R/R DLBCL patients [24]. Furthermore, combining Mosunetuzumab (a CD20xCD3 bispecific) with Pola shows a favorable safety profile and highly durable responses, suggesting promise as second‐line therapy for transplant‐ineligible R/R large B‐cell lymphoma [25]. These findings support the need for further clinical and real‐world studies to optimize combination strategies and enhance outcomes for R/R DLBCL patients.

Patients with R/R DLBCL may benefit from CAR‐T‐cell therapy; however, its application has been limited, as patients with rapidly progressing disease cannot receive timely and enough disease management to proceed to CAR T‐cell treatment [26]. Patients previously exposed to drugs like Bendamustine and Lenalidomide (such as BR or R2 regimens) may also find it more difficult to obtain hematopoietic stem cells or to produce enough CAR‐T cells, especially if they are older than 60 [27]. Therefore, vigilance should be exercised when using regimens that contain Lenalidomide or Bendamustine before mobilization. Although Pola‐BR represents a novel, effective therapeutic regimen for patients with transplantation‐ineligible R/R DLBCL, many patients are either transplant‐eligible or may require CAR‐T cell therapy in the future. In our study, some patients received autologous stem cell transplantation (ASCT) or CAR‐T cell therapy after achieving partial or complete responses, resulting in a longer survival prognosis. These results suggest that Pola‐ZR/G may serve as a bridging therapy for patients awaiting ASCT or CAR‐T therapy as well as a salvage therapy for transplant‐ineligible R/R DLBCL patients.

The small sample size after PSM (20 patients per arm) is a potential limitation of our study. PSM balances observed covariates but cannot account for unmeasured confounders (e.g., socioeconomic factors affecting care access). Observed efficacy differences, especially for survival analysis, which often requires large samples and long follow‐up, could plausibly be influenced by these hidden factors. We did not further analyze differences in effectiveness and safety between Pola‐ZR and Pola‐ZG. Although the effectiveness of the Pola‐G (Obinutuzumab) combination in treating follicular lymphoma has been confirmed [28, 29, 30], there is ongoing debate on the use of these two drugs in R/R DLBCL [28, 31]. In the Phillips et al. [28] research, the R/R DLBCL that received Pola‐G, the investigator‐assessed BOR was 40.9% with a median OS of 10.7 months and a median PFS of 2.8 months (95% CI 1.5–6.3). There was no advantage of Obinutuzumab over Rituximab. However, the Pola‐ZG triple combination outperformed TST in our study, indicating that the Pola‐ZG treatment plan is safe and practical for R/R DLBCL. We anticipate that more prospective randomized controlled clinical trials will validate our finding in the future. The use of the Hans classification and the sample size of COO subgroups may have affected the ability to detect statistical differences. We plan to perform a prospective study of the Pola‐ZR regimen in R/R DLBCL patients because of the positive response of this observational investigation. The protocol was previously registered on ClinicalTrials.gov (NCT05940051).

In conclusion, in a highly refractory high‐risk group of patients with R/R DLBCL, Pola‐ZR/G has shown promising therapeutic improvement with a manageable safety profile.

## Author Contributions

Prof. Peng Liu and Dr. Yian Zhang designed the study. Dr. Yian Zhang contributed to the data analysis and drafting of the article. Dr. Yuhong Ren, Dr. Jingli Zhuang, Dr. Ling Yuan, Dr. Zhimei Wang, Dr. Xuejiao Zhang, Dr. Weiguang Wang, Dr. Zhixiang Cheng, Dr. Luya Cheng, and Dr. Jing Li contributed to data collection and effectiveness and safety assessment. All authors reviewed and approved the final manuscript.

## Ethics Statement

This study was conducted in accordance with the ethical principles of the Declaration of Helsinki and was approved by the ethics committee of the Zhongshan Hospital, Fudan University.

## Consent

All study participants provided informed consent to donate biological samples and health‐related information. The samples and information are anonymized, and will not be shared with independent third‐party research institutes or researchers.

## Conflicts of Interest

The authors declare no conflicts of interest.

## Supporting information


Tables S1–S4:
**Figure S1:** Effectiveness of Pola‐ZR and Pola‐ZG of all treatment lines. BOR, best overall response rate; CRR, complete response rate.

## Data Availability

Deidentified individual participant data that underlie the reported results will be made available 3 months after publication for a period of 5 years upon e‐mail request to corresponding author liu.peng@zs-hospital.sh.cn.

## References

[1] R. Schmitz , G. W. Wright , D. W. Huang , et al., “Genetics and Pathogenesis of Diffuse Large B‐Cell Lymphoma,” New England Journal of Medicine 378, no. 15 (2018): 1396–1407. 29641966 10.1056/NEJMoa1801445 PMC6010183

[2] C. Gisselbrecht , B. Glass , N. Mounier , et al., “Salvage Regimens With Autologous Transplantation for Relapsed Large B‐Cell Lymphoma in the Rituximab Era,” Journal of Clinical Oncology 28, no. 27 (2010): 4184–4190. 20660832 10.1200/JCO.2010.28.1618 PMC3664033

[3] N. Mounier , T. El Gnaoui , H. Tilly , et al., “Rituximab Plus Gemcitabine and Oxaliplatin in Patients With Refractory/Relapsed Diffuse Large B‐Cell Lymphoma Who Are Not Candidates for High‐Dose Therapy. A Phase II Lymphoma Study Association Trial,” Haematologica 98, no. 11 (2013): 1726–1731. 23753028 10.3324/haematol.2013.090597 PMC3815173

[4] G. Salles , J. Duell , E. Gonzalez Barca , et al., “Tafasitamab Plus Lenalidomide in Relapsed or Refractory Diffuse Large B‐Cell Lymphoma (L‐MIND): A Multicentre, Prospective, Single‐Arm, Phase 2 Study,” Lancet Oncology 21, no. 7 (2020): 978–988. 32511983 10.1016/S1470-2045(20)30225-4

[5] L. E. Budde , L. H. Sehn , M. Matasar , et al., “Safety and Efficacy of Mosunetuzumab, a Bispecific Antibody, in Patients With Relapsed or Refractory Follicular Lymphoma: A Single‐Arm, Multicentre, Phase 2 Study,” Lancet Oncology 23, no. 8 (2022): 1055–1065. 35803286 10.1016/S1470-2045(22)00335-7

[6] C. Thieblemont , T. Phillips , H. Ghesquieres , et al., “Epcoritamab, a Novel, Subcutaneous CD3xCD20 Bispecific T‐Cell‐Engaging Antibody, in Relapsed or Refractory Large B‐Cell Lymphoma: Dose Expansion in a Phase I/II Trial,” Journal of Clinical Oncology 41, no. 12 (2023): 2238–2247. 36548927 10.1200/JCO.22.01725 PMC10115554

[7] M. J. Dickinson , C. Carlo‐Stella , F. Morschhauser , et al., “Glofitamab for Relapsed or Refractory Diffuse Large B‐Cell Lymphoma,” New England Journal of Medicine 387, no. 24 (2022): 2220–2231. 36507690 10.1056/NEJMoa2206913

[8] G. Gini , M. Tani , A. Tucci , et al., “Lenalidomide Plus Rituximab for the Initial Treatment of Frail Older Patients With DLBCL: The FIL_ReRi Phase 2 Study,” Blood 142, no. 17 (2023): 1438–1447. 37418685 10.1182/blood.2022019173

[9] H. Scholze , R. E. Stephenson , R. Reynolds , et al., “Combined EZH2 and Bcl‐2 Inhibitors as Precision Therapy for Genetically Defined DLBCL Subtypes,” Blood Advances 4, no. 20 (2020): 5226–5231. 33104794 10.1182/bloodadvances.2020002580 PMC7594393

[10] S. J. Schuster , M. R. Bishop , C. S. Tam , et al., “Tisagenlecleucel in Adult Relapsed or Refractory Diffuse Large B‐Cell Lymphoma,” New England Journal of Medicine 380, no. 1 (2019): 45–56. 30501490 10.1056/NEJMoa1804980

[11] P. Abrisqueta , E. Gonzalez‐Barca , C. Panizo , et al., “Polatuzumab Vedotin Plus Rituximab and Lenalidomide in Patients With Relapsed or Refractory Diffuse Large B‐Cell Lymphoma: A Cohort of a Multicentre, Single‐Arm, Phase 1b/2 Study,” Lancet Haematology 11, no. 2 (2024): e136–e146. 38190832 10.1016/S2352-3026(23)00345-9

[12] L. H. Sehn , A. F. Herrera , C. R. Flowers , et al., “Polatuzumab Vedotin in Relapsed or Refractory Diffuse Large B‐Cell Lymphoma,” Journal of Clinical Oncology 38, no. 2 (2020): 155–165. 31693429 10.1200/JCO.19.00172 PMC7032881

[13] L. H. Sehn , M. Hertzberg , S. Opat , et al., “Polatuzumab Vedotin Plus Bendamustine and Rituximab in Relapsed/Refractory DLBCL: Survival Update and New Extension Cohort Data,” Blood Advances 6, no. 2 (2022): 533–543. 34749395 10.1182/bloodadvances.2021005794 PMC8791582

[14] N. Kawasaki , Y. Nishito , Y. Yoshimura , and S. Yoshiura , “The Molecular Rationale for the Combination of Polatuzumab Vedotin Plus Rituximab in Diffuse Large B‐Cell Lymphoma,” British Journal of Haematology 199, no. 2 (2022): 245–255. 35764309 10.1111/bjh.18341 PMC9796291

[15] Y. Zhu , X. Zhang , J. Wei , et al., “Rituximab, Lenalidomide and BTK Inhibitor as Frontline Treatment for Elderly or Unfit Patients With Diffuse Large B‐Cell Lymphoma: A Real‐World Analysis of Single Center,” Experimental Hematology & Oncology 11, no. 1 (2022): 57. 36114573 10.1186/s40164-022-00314-w PMC9479281

[16] C. Park , H. S. Lee , K. W. Kang , et al., “Combination of Acalabrutinib With Lenalidomide and Rituximab in Relapsed/Refractory Aggressive B‐Cell Non‐Hodgkin Lymphoma: A Single‐Arm Phase II Trial,” Nature Communications 15, no. 1 (2024): 2776. 10.1038/s41467-024-47198-4 PMC10981676 38555311

[17] H. Yu , X. Wang , J. Li , et al., “Addition of BTK Inhibitor Orelabrutinib to Rituximab Improved Anti‐Tumor Effects in B Cell Lymphoma,” Molecular Therapy ‐ Oncolytics 21 (2021): 158–170. 33981831 10.1016/j.omto.2021.03.015 PMC8082047

[18] J. R. Brown , B. Eichhorst , P. Hillmen , et al., “Zanubrutinib or Ibrutinib in Relapsed or Refractory Chronic Lymphocytic Leukemia,” New England Journal of Medicine 388, no. 4 (2023): 319–332. 36511784 10.1056/NEJMoa2211582

[19] C. S. Tam , S. Opat , S. D'Sa , et al., “A Randomized Phase 3 Trial of Zanubrutinib vs Ibrutinib in Symptomatic Waldenstrom Macroglobulinemia: The ASPEN Study,” Blood 136, no. 18 (2020): 2038–2050. 32731259 10.1182/blood.2020006844 PMC7596850

[20] M. A. Dimopoulos , S. Opat , S. D'Sa , et al., “Zanubrutinib Versus Ibrutinib in Symptomatic Waldenstrom Macroglobulinemia: Final Analysis From the Randomized Phase III ASPEN Study,” Journal of Clinical Oncology 41, no. 33 (2023): 5099–5106. 37478390 10.1200/JCO.22.02830 PMC10666987

[21] A. L. Gagez and G. Cartron , “Obinutuzumab: A New Class of Anti‐CD20 Monoclonal Antibody,” Current Opinion in Oncology 26, no. 5 (2014): 484–491. 25014645 10.1097/CCO.0000000000000107

[22] F. L. Locke , D. B. Miklos , C. A. Jacobson , et al., “Axicabtagene Ciloleucel as Second‐Line Therapy for Large B‐Cell Lymphoma,” New England Journal of Medicine 386, no. 7 (2022): 640–654. 34891224 10.1056/NEJMoa2116133

[23] Y. W. Wang , X. C. Tsai , H. A. Hou , et al., “Polatuzumab Vedotin‐Based Salvage Immunochemotherapy as Third‐Line or Beyond Treatment for Patients With Diffuse Large B‐Cell Lymphoma: A Real‐World Experience,” Annals of Hematology 101, no. 2 (2022): 349–358. 34766217 10.1007/s00277-021-04711-9

[24] J. S. Abramson , M. Ku , M. Hertzberg , et al., “Glofitamab Plus Gemcitabine and Oxaliplatin (GemOx) Versus Rituximab‐GemOx for Relapsed or Refractory Diffuse Large B‐Cell Lymphoma (STARGLO): A Global Phase 3, Randomised, Open‐Label Trial,” Lancet 404, no. 10466 (2024): 1940–1954. 39550172 10.1016/S0140-6736(24)01774-4

[25] L. E. Budde , A. J. Olszewski , S. Assouline , et al., “Mosunetuzumab With Polatuzumab Vedotin in Relapsed or Refractory Aggressive Large B Cell Lymphoma: A Phase 1b/2 Trial,” Nature Medicine 30, no. 1 (2024): 229–239. 10.1038/s41591-023-02726-5 PMC10803244 38072960

[26] T. Jo , S. Yoshihara , Y. Okuyama , et al., “Risk Factors for CAR‐T Cell Manufacturing Failure Among DLBCL Patients: A Nationwide Survey in Japan,” British Journal of Haematology 202, no. 2 (2023): 256–266. 37096915 10.1111/bjh.18831

[27] G. Iacoboni , V. Navarro , A. A. Martin‐Lopez , et al., “Recent Bendamustine Treatment Before Apheresis Has a Negative Impact on Outcomes in Patients With Large B‐Cell Lymphoma Receiving Chimeric Antigen Receptor T‐Cell Therapy,” Journal of Clinical Oncology 42, no. 2 (2024): 205–217. 37874957 10.1200/JCO.23.01097

[28] T. Phillips , M. Brunvand , A. I. Chen , et al., “Safety and Efficacy of Polatuzumab Vedotin + Obinutuzumab for Relapsed/Refractory Non‐Hodgkin Lymphomas: A Phase IB/II Study,” American Journal of Hematology 97, no. 1 (2022): E24–E27. 34731510 10.1002/ajh.26400

[29] C. Diefenbach , B. S. Kahl , A. McMillan , et al., “Polatuzumab Vedotin Plus Obinutuzumab and Lenalidomide in Patients With Relapsed or Refractory Follicular Lymphoma: A Cohort of a Multicentre, Single‐Arm, Phase 1b/2 Study,” Lancet Haematology 8, no. 12 (2021): e891–e901. 34826412 10.1016/S2352-3026(21)00311-2

[30] C. R. Flowers , M. J. Matasar , A. F. Herrera , et al., “Polatuzumab Vedotin Plus Bendamustine and Rituximab or Obinutuzumab in Relapsed/Refractory Follicular Lymphoma: A Phase Ib/II Study,” Haematologica 109, no. 4 (2024): 1194–1205. 37767550 10.3324/haematol.2023.283557 PMC10985435

[31] S. Yuen , T. J. Phillips , R. Bannerji , et al., “Polatuzumab Vedotin, Venetoclax, and an Anti‐CD20 Monoclonal Antibody in Relapsed/Refractory B‐Cell Non‐Hodgkin Lymphoma,” American Journal of Hematology 99, no. 7 (2024): 1281–1289. 38700035 10.1002/ajh.27341

