# Supplementary material for: Effectiveness and Safety of Polatuzumab Vedotin Plus an Anti‐CD20 Monoclonal Antibody (Rituximab or Obinutuzumab) and Zanubrutinib in Relapsed/Refractory Diffuse Large B‐Cell Lymphoma
Source: Cancer Med. 2025 Aug 19;14(16):e71162. doi: 10.1002/cam4.71162 (PMC12361929; doi:10.1002/cam4.71162)

**supplementary information**

**Supplementary Tables**

**Table S1. Effectiveness of Pola-ZR/G of different treatment lines**

|  | **2nd Line**  Total  (n=12) | Pola-ZR  (n=6) | Pola-ZG  (n=6) | **≥3rd Line**  Total  (n=8) | Pola-ZR (n=3) | Pola-ZG  (n=5) |
| --- | --- | --- | --- | --- | --- | --- |
| **BOR** | 66.67% (8/12) | 66.67% (4/6) | 66.67% (4/6) | 75%  (6/8) | 100%  (3/3) | 60%  (3/5) |
| **CRR** | 41.67% (5/12) | 33.33% (2/6) | 50% (3/6) | 50%  (4/8) | 66.67% (2/3) | 40%  (2/5) |

BOR, best overall response rate; CRR, complete response rate; Pola-ZR, Polatuzumab vedotin, Zanubrutinib and Rituximab;Pola-ZG, Polatuzumab vedotin, Zanubrutinib and Obinutuzumab;

**Table S2. Clinical characteristics of 1:1 PSM patients**

|  | **Before PSM** | |  | **After PSM** | |  |
| --- | --- | --- | --- | --- | --- | --- |
|  | **Pola-ZR/G group (n = 20)** | **TST group**  **(n =73)** |  | **Pola-ZR/G group (n = 20)** | **TST group**  **(n =20)** |  |
|  | **n (%)** | **n (%)** | ***P*-value** | **n (%)** | **n (%)** | ***P*-value** |
| **Median age, years (range)** | 68 | 60 | **0.045** | 68 | 67.5 | 0.778 |
| **Age≥70, n (%)** | 9(45%) | 19(26.9%) | 0.101 | 9(45%) | 8(40%) | 0.749 |
| **Male, n (%)** | 13(65%) | 42(57.53%) | 0.547 | 13(65%) | 14(70%) | 1 |
| **PS≥2, n (%)** | 14(70%) | 34(46.6%) | 0.063 | 14(70%) | 11(55%) | 0.327 |
| **Ann Arbor stage, n (%)** |  | | | | | |
| I-II | 2(10%) | 15(20.5%) | 0.348 | 2(10%) | 3(15%) | 1 |
| ≥III | 18(90%) | 58(79.5) |  | 18(90%) | 17(85%) |  |
| **IPI score, n (%)** |  |  |  |  |  |  |
| 0-2 | 4(20%) | 31(42.5%) | 0.066 | 4(20%) | 3(15%) | 1 |
| 3-5 | 16(80%) | 42(57.5) |  | 16(80%) | 17(85%) |  |
| **COO, n (%)** |  | | | | | |
| GCB | 9(45%) | 24(32.9%) | 0.315 | 9(45%) | 7(35%) | 0.519 |
| nonGCB | 11(55%) | 49(67.1%) |  | 11(55%) | 13(65%) |  |
| **Bulky disease (≥7 cm), n (%)** | 8(40%) | 23(31.5%) | 0.475 | 8(40%) | 9(45%) | 0.749 |
| **DHL or DEL, n (%)** |  |  |  |  |  |  |
| Yes | 9(45%) | 11(15.1%) | **0.011** | 9(45%) | 6(30%) | 0.327 |
| No | 11(55%) | 62(84.9%) |  | 11(55%) | 14(70%) |  |

PSM, propensity score matching; TST, traditional salvage therapies; DHL, double hit lymphoma; DEL, double expression lymphoma; Pola-ZR, Polatuzumab vedotin, Zanubrutinib and Rituximab;Pola-ZG, Polatuzumab vedotin, Zanubrutinib and Obinutuzumab; PS, performance score; IPI, international prognostic index; COO, cell of origin; GCB, germinal center B-cell like;

**Table S3. Treatment response before and after 1:1 PSM**

|  |  | **Before PSM** | |  | **After PSM** | |  |
| --- | --- | --- | --- | --- | --- | --- | --- |
| **No. of Line** |  | **Pola-ZR/G**  **(n = 20)** | **TST**  **(n =73)** | ***P*-value** | **Pola-ZR/G**  **(n = 20)** | **TST (n =20 )** | ***P*-value** |
| **Any-Line** | BOR | 70% (14/20) | 34.2% (25/73) | **0.004** | 70% (14/20) | 35% (7/20) | **0.027** |
|  | CRR | 45% (9/20) | 16.4% (12/73) | **0.016** | 45% (9/20) | 10% (2/20) | **0.013** |
| **2nd Line** | BOR | 66.7% (8/12) | 39.3% (24/61) | 0.153 | 66.7% (8/12) | 38.9% (7/18) | 0.136 |
|  | CRR | 41.7% (5/12) | 18% (11/61) | 0.12 | 41.7% (5/12) | 11.1% (2/18) | 0.084 |
| **≥3rd Line** | BOR | 75% (6/8) | 8.3% (1/12) | **0.004** | 75% (6/8) | 0% | 0.133 |
|  | CRR | 50% (4/8) | 8.3% (1/12) | 0.109 | 50% (4/8) | 0% | 0.467 |

BOR, best overall response rate; CRR, complete response rate; Pola-ZR, Polatuzumab vedotin, Zanubrutinib and Rituximab;

Pola-ZG, Polatuzumab vedotin, Zanubrutinib and Obinutuzumab; TST, traditional salvage therapies; PSM, propensity score matching

**Table S4. Summary of the Polatuzumab vedotin-based studies for R/R DLBCL**

| **Author** | **Year** | **Therapy** | **Trial phase** | | **Patient number** | **Median age** | **ORR** | **CRR** |  |
| --- | --- | --- | --- | --- | --- | --- | --- | --- | --- |
| **Our study Liu et al** | 2024 | Pola-ZR and Pola-ZG | prospective observational cohort study | | 22 | 68 | BOR Overall：70% | CRR Overall：45% |  |
|  |  |  |  |  |  |  | 2nd Line：66.7% | 2nd Line: 41.7% |  |
|  |  |  |  |  |  |  | ≥3rd Line: 75% | ≥3rd Line: 50% |  |
| **Pau Abrisqueta et al** | 2024 | Pola+Rituximab+Lenalidomide（GO29834） | Phase 2 | | 39 | 71 | 46% (IRC) BOR：74% | 39%(IRC) BCR：37% |  |
|  |  |  |  |  |  |  |  |  |  |
| **Yasuhito Terui et al** | 2023 | Pola-BR | Phase 2 | | 35 | 71 | 42.9% at EOT | 34.3% at EOT |  |
| **Laurie H Sehn et al** | 2022 | Pola-BR (GO29365) | Phase 1b/2 (extension cohort) | | 106 | 67 | BOR: 56.6% (IRC)  62.3 (INV) | BCR: 52.8% (IRC)  50% (INV) |  |
|  |  |  | Phase 1b/2 | 40 | | 70 | BOR: 62.5% (IRC)  70% (INV) | BCR: 52.5% (IRC)  57.5% (INV) |  |
| **Yu‑Wen Wang et al** | 2022 | Pola-Rituximab /Rituximab + Bendamustine/ Rituximab + Gemcitabine-based/ Rituximab + Carmustine-based | Retrospective | | 40 | 59 | 52.50% | 25% |  |
| **AMinoGhobadi et al** | 2021 | Axi-cell (Zuma7 study) | Phase 3 | | 180 | Axi-cell：58 | 2nd Line CAR-T:83% | 2nd Line CAR-T: 65% |  |
|  |  |  |  |  |  | SOC：60 | 3rd Line CAR-T :57% | 3rd Line CAR-T :34% |  |
|  |  |  |  |  |  |  | 2nd Line SOC: 50% | 2nd Line SOC: 32% |  |

ORR, overall response rate; BOR, best overall response rate; CRR, complete response rate; BCR, best complete response rate; Pola-ZR, Polatuzumab vedotin, Zanubrutinib and Rituximab; Pola-ZG, Polatuzumab vedotin, Zanubrutinib and Obinutuzumab; IRC, Independent Review Committee; INV, investigator; EOT, end of treatment; SOC, stand of care

**Supplementary Figure Legend**

**Figure S1. Effectiveness of Pola-ZR and Pola-ZG of all treatment lines**

BOR, best overall response rate; CRR, complete response rate


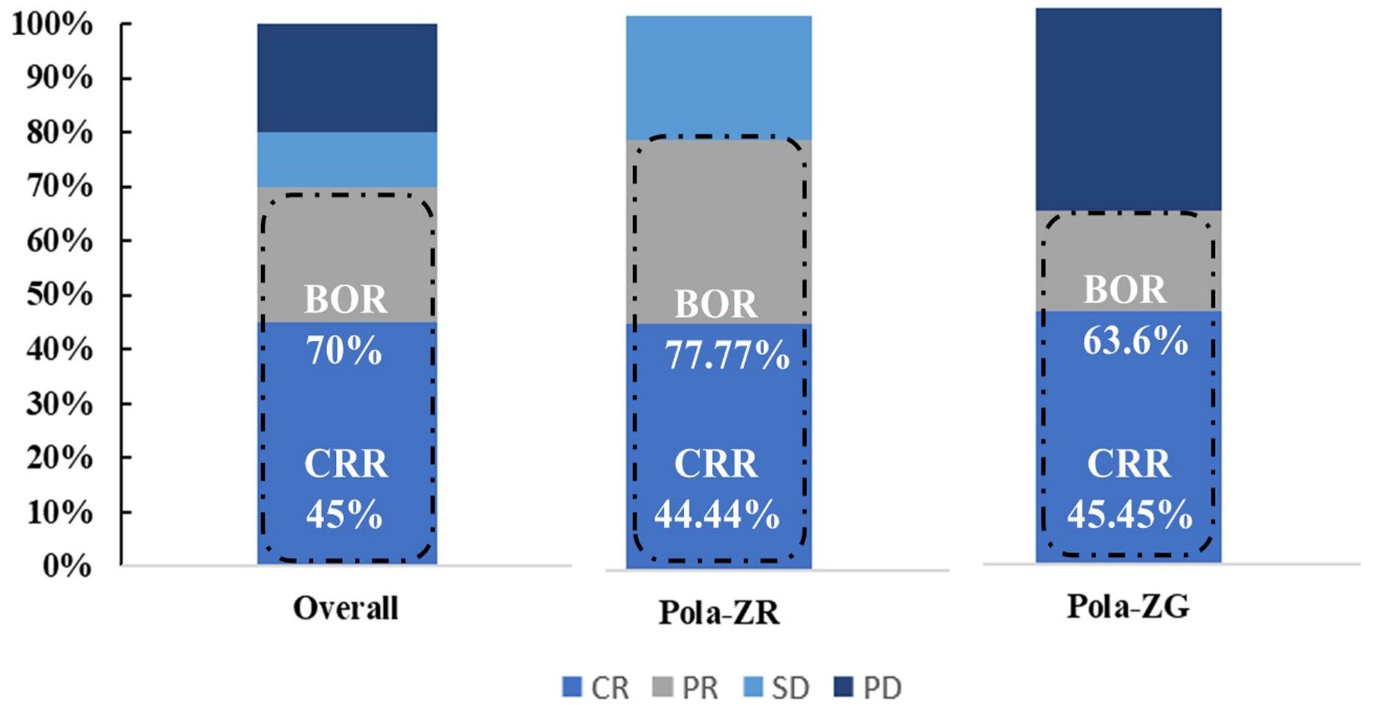

Supplement: Supplementary file 1 — Tables S1–S4: Figure S1: Effectiveness of Pola‐ZR and Pola‐ZG of all treatment lines. BOR, best overall response rate; CRR, complete response rate. [file CAM4-14-e71162-s001.docx]
